# Supplementary material for: Cognitive Sparing in Proton versus Photon Radiotherapy for Pediatric Brain Tumor Is Associated with White Matter Integrity: An Exploratory Study
Source: Cancers (Basel). 2023 Mar 19;15(6):1844. doi: 10.3390/cancers15061844 (PMC10047305; doi:10.3390/cancers15061844)
Supplement: Supplementary file 1 [file cancers-15-01844-s001.zip › cancers-2219510-supplementary.pdf]

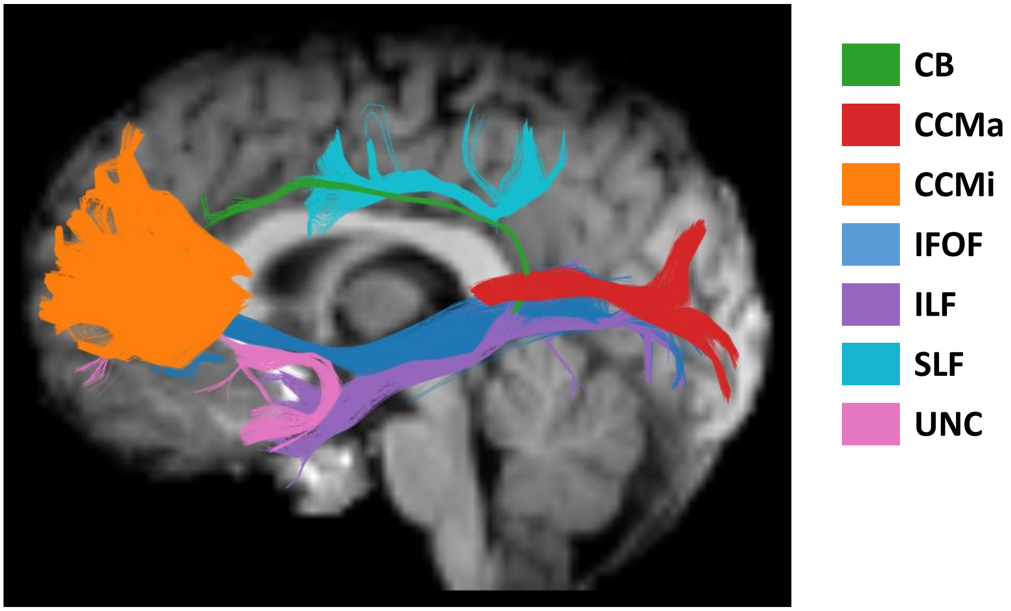

Figure S1. Color-coded tracts of interest are shown for a 12-year-old male participant in the control group. Cingulum bundle (CB); Corpus callosum major forceps (CCMa); Corpus callosum minor forceps (CCMi); Inferior fronto-occipital fasciculus (IFOF); Inferior longitudinal fasciculus (ILF); Superior longitudinal fasciculus (SLF); Uncinate fasciculus (UNC)

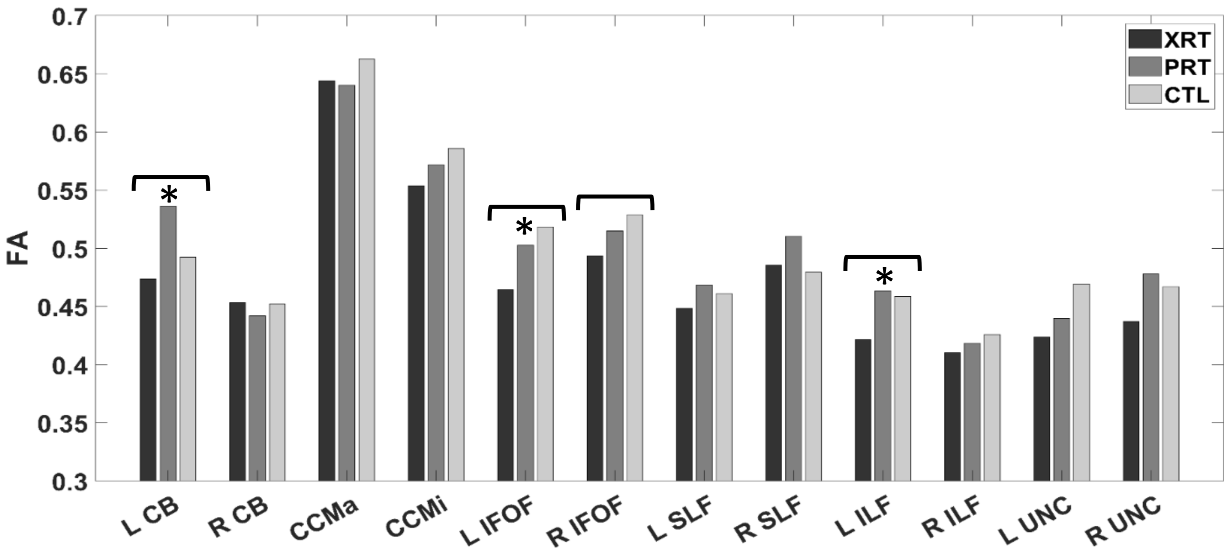

Figure S2. Unadjusted FA group means are shown for each tract for focal radiotherapy patients only. Group differences were determined by ANCOVA with age and handedness as covariates, with α = .05. Significant omnibus group differences (XRT-PRT-CTL) are depicted by brackets. Significant XRT-PRT differences are depicted by *.

Table S1. Tumor Types and Locations for XRT and PRT Patients

Patient ID	Group	Tumor Type	Primary Site	Other Tumor Sites	Laterality
PRT01	PRT	Medulloblastoma	4th ventricle, arises from vermis	---	---
PRT02	PRT	Medulloblastoma	Posterior fossa	Extension through foramen of Luschka	---
PRT03	PRT	Medulloblastoma	Cerebellum	Extension into right cerebellopontine angle cistern; Extends along right cisternal nerves 7 and 8 within cerebellopontine angle	---
PRT04	PRT	Pilocytic astrocytoma	Left thalamus	---	Left
PRT05	PRT	Anaplastic ependymoma	Frontal lobe	---	Left
PRT06	PRT	Pilomyxoid astrocytoma (Grade II)	Hypothalamus/suprasellar	Extension into 3rd ventricle	---
PRT07	PRT	Pilocytic astrocytoma	4th ventricle	Cystic component left of midline; Left middle cerebellar peduncle	---
PRT08*	PRT	Medulloblastoma	Posterior fossa	---	---
PRT09	PRT	Craniopharyngioma	Hypothalamus/suprasellar	Impinging on 3rd ventricle	---
PRT10	PRT	Pilocytic astrocytoma	Hypothalamus/suprasellar	Extends into 3rd ventricle	---
PRT11	PRT	Desmoplastic ganglioglioma	Left basal ganglia; Left cerebral hemisphere	Extends into insula and left frontal and temporal lobes	Left
PRT12	PRT	Anaplastic ependymoma	Anterior vermis	Cystic loculation in left cerebellar medullary angle; Extends into 4th ventricle; Growth into bilateral foramina Luschka and through foramen of Magendie; Extends along posterior aspect of cervical medullary junction to level of posterior C1 canal; Extension into left cerebellar medullary angle	---
XRT01*	XRT	Pilocytic astrocytoma	Cerebellum; Brainstem/pons	---	---
XRT02	XRT	Medulloblastoma	4th ventricle	Extension into aqueduct;	---

				Extension into cerebello-medullary angle through left foramen of Luschka	
XRT03	XRT	Choroid plexus carcinoma	Posterior horn of the right lateral ventricle	---	Right
XRT04*	XRT	Pilocytic astrocytoma	Hypothalamus/suprasellar	---	---
XRT05	XRT	Medulloblastoma	Posterior fossa anterior to vermis; filling 4th ventricle	Extension down through the plane with tonsils at the level of the obex; Partial extension into left foramen of Luschka.	---
XRT06	XRT	Atypical choroid plexus tumor	Posterior fossa;<br>Cerebellar pontine angle cistern;<br>Foramen of Luschka	Extends through foramen magnum	---
XRT07	XRT	Primitive neuroectodermal tumor- Not otherwise specified	Frontal lobe	---	Left
XRT08	XRT	Anaplastic ependymoma	Posterior fossa	Extension of tumor to C3-C4	---
XRT09	XRT	Medulloblastoma	4th ventricle	Extension into foramen of Magendie;<br>Extension behind medulla and upper spinal cord;<br>Extension into upper spinal canal	---
XRT10	XRT	High grade neoplasm with small blue cell features	Frontal lobe;<br>Temporal lobe;<br>Hypothalamus/suprasellar	---	Right

[1]Tumor locations are based on each patient's first MRI report prior to surgical resection. Three patients (denoted by *) had this MRI at an outside hospital. Outside MRI records were reviewed by a neuro-oncologist who determined tumor location.

Table S2. Results of Linear Mixed Models: Focal Radiotherapy Patients Only

	XRT (n = 5) vs. PRT (n = 8)			PRT (n = 8) vs. CTL (n = 23)		
	β	*t*	*p*	*β*	*t*	*p*
FA	-0.034	-2.47	**.014**	0.007	.706	.480
AD	-0.023	-0.87	.380	-0.009	-0.52	.603
RD	0.036	3.22	**.001**	-0.011	-1.41	.159

[1]*β, t, and* - and *p*-values are derived from linear mixed models with handedness and age as fixed covariates and subject as a random variable.

[2]**Bold text** indicates $p < .05$.

Table S3. Neuropsychological Test Scores: Focal Radiotherapy Patients Only

	**XRT (*n*=5)**		**PRT (*n*=8)**		**CTL (*n* =23)**	
	Mean(SD)	*Min-Max*	*Mean(SD)*	*Min-Max*	*Mean(SD)*	*Min-Max*
FSIQ	89.4 (12.9)	72 – 106	104.4 (11.5)	93 – 129	99.7 (10.9)	84 - 125
VCI	88.0 (11.7)	78 – 107	107.8 (13.7)	92 – 136	98.1 (12.8)	78 – 118
PRI	98.4 (19.0)	75 – 119	108.3 (9.6)	96 – 125	100.0 (12.3)	79 – 125
WMI	94.6 (7.4)	85 – 105	104.5 (14.0)	92 – 135	102.2 (10.3)	88 – 122
PSI	82.8 (20.3)	60 – 103	94.3 (18.2)	72 – 126	101.0 (11.2)	75 – 123
VMI	73.2 (21.3)	50 – 103	95.0 (9.3)	82 – 112	86.5 (11.5)	50 – 104
MC	60.8 (14.4)	45 – 72	88.4 (12.9)	71 – 102	88.1 (11.4)	61 – 102

	**XRT vs. PRT**		**PRT vs. CTL**	
	t	*p*	*t*	*p*
FSIQ	-2.57	**0.015**	-1.08	0.290
VCI	-2.60	**0.014**	-1.37	0.181
PRI	-1.46	0.155	-1.15	0.259
WMI	-1.71	0.097	-0.90	0.374
PSI	-1.67	0.106	0.36	0.721
VMI	-2.23	**0.033**	-1.48	0.149
MC	-4.71	**<.001**	-0.84	0.405

[1]Unadjusted means are presented. *t*- and *p*-values are derived from ANCOVAs with handedness and age as covariates.

[2]**Bold text** indicates $p < .05$.
